# Delayed diagnosis of Swyer‐James‐MacLeod syndrome

**DOI:** 10.1002/rcr2.1382

**Published:** 2024-05-17

**Authors:** Matthew T. Donnan, Eli Dabscheck, Belinda R. Miller, Samantha J. Ellis, Matthew T. Naughton

**Affiliations:** ^1^ Department of Respiratory Medicine The Alfred Hospital Melbourne Victoria Australia; ^2^ Central Clinical School Monash University, The Alfred Hospital Melbourne Victoria Australia; ^3^ Department of Radiology The Alfred Hospital Melbourne Victoria Australia; ^4^ Department of Surgery Monash University, The Alfred Hospital Melbourne Victoria Australia

**Keywords:** computed tomography, congenital, developmental, Swyer‐James‐MacLeod syndrome

## Abstract

Swyer‐James‐MacLeod Syndrome is a rare obliterative lung disease typically caused by childhood infection resulting in arrested pulmonary development. Imaging findings include unilateral hyperlucency on chest x‐ray, and hyperlucency, hypovascularity and expiratory gas trapping on computed tomography. Recognition of abnormal imaging can lead to earlier diagnosis and institution of appropriate management.

## Graphical abstract

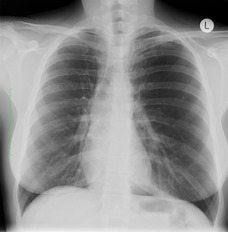

## CLINICAL IMAGE

**FIGURE 1:**
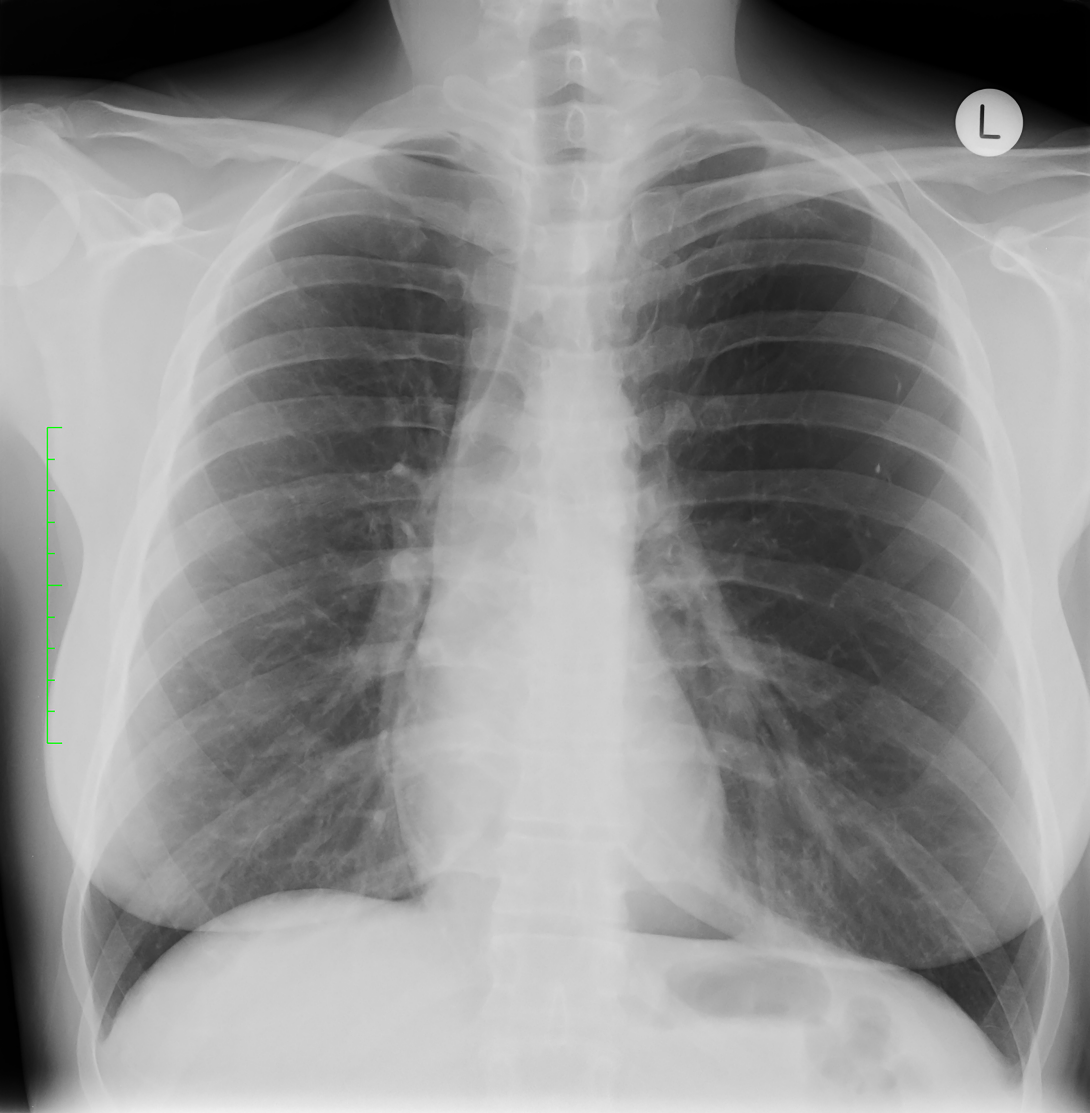
Plain chest x‐ray demonstrating left sided hyperlucency and mediastinal shift.

**FIGURE 2:**
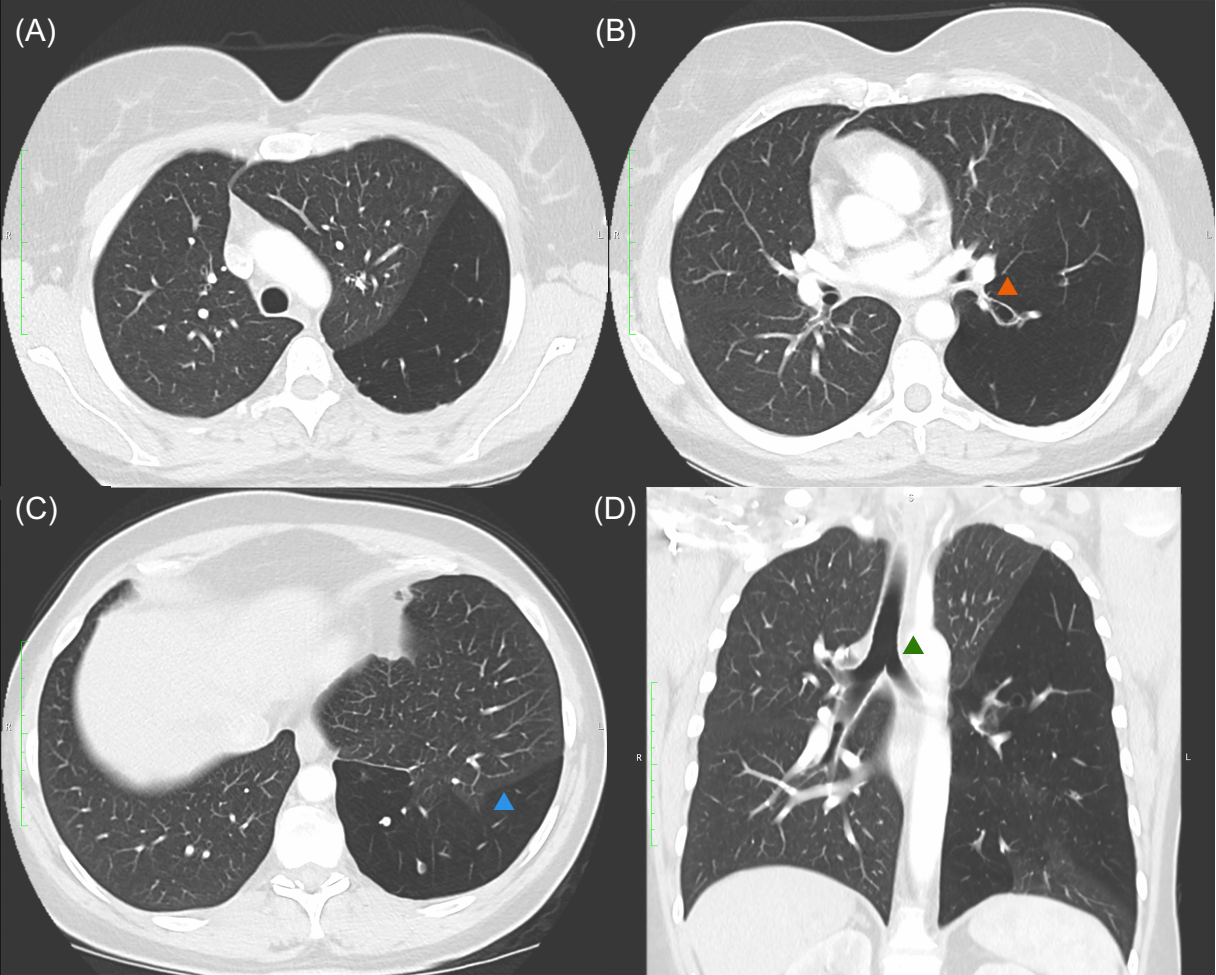
Sagittal and coronal computed tomography images (clockwise from top left; A–D) showing left lower lobe hyperlucency and hypovascularity. (A) demonstrates hyperlucency and hypovascularity of the left lower lobe. (B) demonstrates stenosis at the origin of the left lower lobe bronchus with distal ballooning (orange arrow). (C) demonstrates an incomplete oblique fissure and subsequent parenchymal intrusion into the left lower lobe (blue arrow). In SJMS the volume of the affected lung is normal or, more commonly, reduced. It is seldom if ever increased. In this case collateral air drift through the incomplete fissure is the likely cause of the hyperinflation and subsequent mass effect. (D) demonstrates the degree of mediastinal shift with rightward tracheal deviation (green arrow). Hyperinflation is not typically a feature of SJMS and is often more suggestive of congenital lobar emphysema. If hyperinflation is present, this suggests presence of collateral air flow either from adjacent unaffected lung, or across an incomplete fissure as seen in this case.

A 51‐year‐old woman was referred to respiratory outpatients with 2 years of chronic productive cough. She was a never smoker but had recurrent respiratory infections during childhood, and pneumonia requiring hospitalization in her 20's. Computed tomography (CT) of the chest revealed hyperlucency, hypovascularity and bronchiectasis of the left lower lobe, with stenosis of the left lower lobe bronchus giving rise to Swyer‐James‐MacLeod Syndrome (SJMS). Hyperinflation with mediastinal shift was noted, with an incomplete oblique fissure and associated parenchymal intrusion of the left upper lobe into the left lower lobe. Chest x‐ray 10 years prior revealed left sided hyperlucency with mediastinal shift; no follow‐up had occurred (Figures 1 and 2). Respiratory function tests revealed an obstructive defect (FEV_1_/FVC 0.59) with an FEV_1_ 2.37 L (84% predicted), FVC 4.04 L (114%), and TLCO 23.76 mL/mmHg/min (113%). 

SJMS is a rare obliterative lung disease typically caused by childhood infection resulting in arrested pulmonary development.^1^ Classical CT findings of SJMS include unilateral hyperlucency, hypovascularity and gas trapping on expiratory films.^2^ Differential diagnoses include an obstructing endobronchial lesion and congenital lobar emphysema. In order not to delay diagnosis and institute appropriate management, an abnormal chest x‐ray should be followed by CT imaging and consideration of bronchoscopy to exclude an endobronchial lesion.^2^

## AUTHOR CONTRIBUTIONS

All authors were involved in patient care. MTD and SE were involved in preparation of the manuscript and images. All authors approved the final manuscript.

## CONFLICT OF INTEREST STATEMENT

None declared.

## ETHICS STATEMENT

The authors declare that appropriate written informed consent was obtained for the publication of this manuscript and accompanying images.

## Data Availability

Data sharing not applicable to this article as no datasets were generated or analysed during the current study.
